# Predicting Academic Achievement with Cognitive Abilities: Cross-Sectional Study across School Education

**DOI:** 10.3390/bs10100158

**Published:** 2020-10-17

**Authors:** Tatiana Tikhomirova, Artem Malykh, Sergey Malykh

**Affiliations:** 1 Department of Psychology, Lomonosov Moscow State University, 125009 Moscow, Russia; tikho@mail.ru; 2 Psychological Institute of Russian Academy of Education, 125009 Moscow, Russia; 3 Russian Academy of Education, 119121 Moscow, Russia; malykhartem86@gmail.com

**Keywords:** academic achievement, cognitive ability, information processing speed, visuospatial working memory, number sense, fluid intelligence, schooling, cross-sectional study

## Abstract

The relationship between cognitive abilities and academic achievement across schooling from the first to the eleventh grade was analyzed. Information processing speed, visuospatial working memory, number sense, and fluid intelligence were considered predictors of general academic achievement, which was derived from grades in mathematics, language, and biology. This cross-sectional study involved 1560 pupils who were in grades 1–11 at general education schools and were aged from 6.8 to 19.1 years (50.4% were boys). Information processing speed, visuospatial working memory, and number sense were measured using the Choice Reaction Time, Corsi Block-Tapping, and Number Sense computerized tests, respectively. Fluid intelligence was measured using the paper-and-pencil version of the Standard Progressive Matrices test. Correlation analysis and structural equation modeling were carried out. It was shown that it is possible to describe the structure of the relationship between cognitive abilities and academic achievement for all levels of schooling with a single model. In this model, information processing speed is the key predictor of fluid intelligence, working memory, and number sense, which in turn contribute to individual differences in academic success. Additionally, the specificity of the relationship between individual indicators of cognitive abilities and academic achievement at each level of schooling was revealed.

## 1. Introduction

The task of predicting academic achievement has been relevant for more than a century, and during this time, various psychological constructs have been analyzed as predictors of academic success [1,2,3,4].

Studies have shown that academic achievement is largely related to the individual characteristics of basic cognitive processes, i.e., information processing speed, visuospatial working memory, and number sense [2,5,6,7,8], as well as higher-order cognitive processes, such as primarily fluid intelligence [1,3,5,9,10].

Information processing speed is the ability to accurately and quickly process incoming information and underlies individual differences in academic success [2,11] and higher-order cognitive abilities [12,13]. A direct relationship with academic achievement [14] and the absence of this relationship [15] have been shown to be indicators of information processing speed at certain ages, and theoretical models have been built in which information processing speed influences higher-order cognitions, which in turn influence individual differences in academic outcomes [10,13,16,17].

Visuospatial working memory is responsible for storing and processing visual information related to spatial positioning and visual stimuli observed during direct perception or extracted from long-term memory [18,19]. Visuospatial working memory is a significant predictor of academic success in virtually all areas of scientific knowledge, from mother-tongue acquisition [1,20] to mathematics [19,21]. Additionally, it has been shown that working memory can be related to different aspects of mathematical knowledge, including understanding the concepts of basic arithmetic operations, complex mathematical calculations, and spatial relations in geometry [7].

Number sense is understood as the ability to estimate the number of objects without counting them directly [4]. Indicators of number sense, which is related to both the symbolic and nonsymbolic evaluation of quantities, are related to academic success in different ways. According to one study, number sense is a reliable predictor of test-based and expert-based indicators of success in mathematical learning from primary to high school [4,15]. In particular, number sense has been shown to exhibit moderate correlations with grades in mathematical education at primary and secondary school education levels [15,22]. In other studies, the relationship between number sense and mathematical achievement was confirmed only in a sample of primary school age students [23,24].

Fluid intelligence is understood as the ability to effectively solve new problems based on the non-verbal abstract reasoning and to successfully adapt to changing environmental conditions. Unlike crystallized intelligence, which directly depends on the individual’s store of knowledge, fluid intelligence depends little on personally acquired knowledge and does not depend on the ability to learn [25]. Fluid intelligence is one of the most significant predictors of individual achievements in various academic fields, as confirmed by studies carried out in various social and economic contexts, educational conditions, and age ranges [1,3,8,10]. These studies and meta-analyses provide data on the strong correlation between fluid intelligence and academic success in various disciplines, with correlation coefficients ranging from 0.37 to 0.63 [5,26]. Moreover, intelligence measured in primary school age has been shown to explain almost 60% of the variance in math test scores during the whole schooling period [3].

In general, basic cognitive traits, along with intelligence, explain up to 60% of the dispersion of academic success [5]. Additionally, studies report that the indicators of cognitive abilities are age-related and have unique developmental trajectories across schooling [6,27,28]. Thus, the trajectory of the development of information processing speed in childhood and adolescence is best described by quadratic dependency, showing an increase in the information processing speed indicators between the ages of 5 and 18, with periods of sharp increases and periods of relative stability [29,30]. Studies of working memory reveal a linear trajectory of working memory development from an early age, indicating that it reaches a peak only at the age of thirty [31,32,33]. The dynamics of change of the number sense, which affects the ability to estimate nonsymbolic quantities, is characterized by relatively slow growth throughout schooling and a lower impact of the educational process on its development [28]. The trajectory of fluid intelligence development is characterized by high stability from early adolescence to late adulthood and highly unequal development from infancy to adolescence, with marked individual differences [34,35].

The results show that the relationship between different cognitive abilities and academic achievement may change with age [36,37,38,39]. A number of studies have shown that a stronger correlation is shown for children compared with adolescents and young adults [38]. However, according to a meta-analysis of studies on the relationship between cognitive development and educational achievement, these relations increase with age, even after controlling for the socioeconomic status [37]. According to cross-cultural studies, the relationship between cognitive development and academic achievement is largely associated with the structure and quality of national school education [6,25,40]. In particular, it was shown that the role of cognitive resources in academic success at school increases in a less heterogeneous and more effective educational environment [6,40].

These data require special consideration of the relationship between cognitive abilities and academic achievement at different schooling periods, which may explain the ambiguous, often diametrically opposed, results observed in studies involving different age and cross-cultural groups of respondents.

The purpose of this study was to examine the relationship between cognitive abilities and academic success throughout schooling, from primary to high levels of school education in the Russian Federation. Information processing speed, visuospatial working memory, number sense, and fluid intelligence were considered as predictors of general academic achievement based on school grades in mathematics, language, and biology.

## 2. Materials and Methods

### 2.1. Participants

This study involved 1560 pupils who were in grades 1–11 in general schools and were aged from 6.8 to 19.1 years (50.4% were boys). There were 876 students in the primary school age sample (grades 1–4, primary level of school education, aged from 6.8 to 11.7 years; 52.6% were boys), 544 students in the secondary general education sample (grades 5–9, secondary level of school education, aged 10.8 to 16.8 years; 54.8% were boys), and 203 students in the high general education sample (grades 10–11, high level of school education, range from 15.3 to 18.8 years; 37.4% were boys).

All subjects gave their informed consent for inclusion before they participated in the study. Parental informed consent was obtained for all participants. The study was conducted in accordance with the Declaration of Helsinki, and the protocol was approved by the Ethics Committee of the Psychological Institute of the Russian Academy of Education (project identification code 2016/2–12). Personal data were anonymized.

### 2.2. Measures

The experiment was performed in a computer classroom in groups of under 15 schoolchildren. Each participant sat in front of their own monitor screen and performed the experiment independently. Each computer had a 17” LCD display with a resolution of 1440 × 900 pixels and a refresh rate of 60 Hz. The participants were seated approximately 60 cm from the screen. Each participant performed the Dot Task, the Choice Reaction Time test, and the Sequences tests on a computer, and in the following two days, they performed the paper-and-pencil version of the Standard Progressive Matrices test [18,27,28,41]. The order of the tests was the same for all participants.

#### 2.2.1. Cognitive Abilities

##### Choice Reaction Time Test, Information Processing Speed

In this test, the numbers 1, 2, 3, and 4 appear 10 times each in a randomized order with random intervals ranging from 1 to 3 s. The test task consists of pressing the key corresponding to the number appearing on the screen as fast and as accurately as possible. The maximum time for responses is 8 s. If no response is given during this time, the next part of the test appears on the screen. The program records the accuracy and reaction time of correct responses. Information processing speed is measured as reaction times. Lower reaction times correspond to higher processing speeds.

##### Corsi Tapping-Block Test, Visuospatial Working Memory

In this test, participants are presented with a row of blocks that glow one at a time in a certain order. The task consists of reproducing the correct order by clicking on the blocks with the computer mouse. The test starts with 4 items (or tapping of the blocks) in each sequence. There are 2 sequences at each of the 6 levels, for a total of 12 trials. If participants complete both or just one correct sequence at one level, the first item of the next level is shown. The test is interrupted when both sequences at the same level are reproduced incorrectly. During the presentation of the stimuli, the block glows for 1 s. There is an interval of 1 s between the glowing of the blocks. The program records the number of correct responses and reaction times during correct responses.

#### Dot Task Test, Number Sense

In this test, participants are presented with arrays of yellow and blue dots, mixed together and varying in size and numbers. The task requires judging whether the array contains more yellow or blue dots by pressing the corresponding keys on the keyboard. The stimuli are 150 static pictures with arrays of yellow and blue dots varying between 5 and 21 dots of each color and ratios of the arrays in two colors between 1:3 and 6:7. The presentation order is the same for all participants. The stimulus flashes on the screen for 400 ms, and the maximum response time is 8 s. The test records accuracy and reaction time on correct trials.

##### Standard Progressive Matrices Test, Fluid Intelligence

Fluid intelligence was measured using the paper-and-pencil version of the Standard Progressive Matrices test. The test comprises 5 sets: A, B, C, D, and E. Within each set, there are 12 items that progressively become more difficult; thus, there were 60 tasks in total. There was no discontinuity rule, and the participants completed all tasks. The accuracy was calculated as a sum of correct answers across tests.

#### 2.2.2. Academic Achievement

Quarterly (for pupils of grades 1–9) and semiannual (for pupils of grades 10–11) grades in mathematics (algebra and geometry), Russian language, and biology were used as indicators of academic achievement. The statistical analysis used the arithmetic mean of the quarterly or semiannual grades.

### 2.3. Statistical Approach

During the first stage, the correlation analysis method was used to examine the relationship between cognitive characteristics (information processing speed, visuospatial working memory, number sense, and fluid intelligence) and academic achievement in Russian language, mathematics, and biology. Spearman correlation coefficients were calculated using the IBM SPSS 20.0 package (IBM Corp., Armonk, NY, USA).

During the second stage, the influence of cognitive characteristics on academic success was analyzed using structural equation modeling (OpenMX package, The OpenMx Development Team, Charlottesville, VA, USA). The following criteria were used to evaluate the model’s compliance with empirical data: RMSEA ≤ 0.06; 95% confidence intervals RMSEA_low_ = 0.00 and RMSEA_high_ < 0.08; CFI > 0.95; TLI > 0.90 [42]. During the structural modeling, a number of theoretical models of the influence of cognitive characteristics on academic success at primary, secondary, and high levels of school education were tested.

- Model 1: Cognitive characteristics affect academic success through the latent variable of general cognitive ability “*g*”;
- Model 2: Cognitive characteristics—information processing speed, visuospatial working memory, number sense, and fluid intelligence—contribute to general academic achievement “*e*” (“education”);
- Model 3: Information processing speed is a key predictor of fluid intelligence, working memory, and number sense, which in turn contribute to general academic achievement.

## 3. Results

The study analyzed the following cognitive characteristics: information processing speed, visuospatial working memory, number sense, fluid intelligence, and academic success in Russian language, mathematics, and biology.

Table 1 shows the mean values and standard deviations of the analyzed characteristics during primary (top line), secondary (middle line), and high (bottom line) levels of school education.

Table 1 shows the number of correct answers on the Standard Progressive Matrices, Sequences, and Dot Task tests, which measure fluid intelligence, visuospatial working memory, and number sense, respectively. The possible scores for Standard Progressive Matrices ranged from 0 to 60, those for the Sequences test ranged from 0 to 12, and those for the Number sense test ranged from 0 to 150. For information processing speed, the average values of reaction time to correct answers in seconds for the Choice Reaction Time test are given. A higher average response time value corresponds to lower information processing speed.

The average quarterly or semiannual grades with a minimum of 2 and a maximum of 5 are used as indicators of academic achievement.

According to Table 1, the mean of all cognitive indicators analyzed increases over time, while the mean academic success scores decrease.

### 3.1. Correlation Analysis

The correlation analysis examined the relationship between cognitive abilities and academic success at each level of school education. Table 2 presents Spearman’s correlation coefficients between processing speed, working memory, number sense, fluid intelligence, and academic success in mathematics, language, and biology.

According to Table 2, the strongest correlations between cognitive characteristics and academic success are found at the primary level of education (compared to the secondary and high levels). For example, fluid intelligence is correlated with academic success in Russian language to varying degrees at the primary level (*r =* 0.47; *p* < 0.01), the secondary level (*r =* 0.33; *p* < 0.01), and the high level (*r =* 0.18; *p* < 0.01) of school education. Moreover, it is shown that at the high level of school education for a number of cognitive characteristics, there are no correlations with academic success. Thus, for the number sense and academic success in, for example, mathematics and Russian language, statistically significant correlations exist at the primary and secondary level but not at the high level (*p* > 0.05).

It should be noted that for cognitive characteristics at each level of school education, academic success is most strongly related to fluid intelligence and, to a lesser extent, to number sense and working memory. For information processing speed, no correlation with academic success was found (*p* > 0.05), with the exception of a weak relationship with academic success in mathematics (*r =* −0.09; *p* < 0.05) at the primary school education.

The correlation analysis revealed a moderate correlation between cognitive characteristics (from 0.16 to 0.42) and a high correlation between grades in different disciplines (from 0.72 to 0.92).

### 3.2. Structural Modeling

During the course of structural modeling, it was shown that the tested theoretical Model 1, which assumes the influence of cognitive characteristics on indicators of academic success through the “*g*” factor, and Model 2, based on the existence of a “side-by-side” contribution of cognitive indicators to the general academic achievement “*e*”, do not correspond well to the data at each level of school education (RMSEA > 0.08; CFI < 0.95; TLI < 0.90; χ^2^ significant (*p* < 0.05)).

Model 3—which posits that information processing speed is a key predictor of fluid intelligence, working memory, and number sense, which in turn contribute to general academic success—is the best fit with the data obtained in this study.

Table 3 shows the model fit indicators obtained at the primary, secondary, and high educational levels.

According to Table 3, at the primary, secondary, and high levels of schooling, the tested model of the impact of cognitive abilities on academic success had good fit indices: RMSEA ≤ 0.06; 95% confidence intervals: RMSEA_low_ = 0.00 and RMSEA_high_ < 0.08; CFI > 0.95; TLI > 0.90. The χ^2^ value was not significant (*p* > 0.05), which indicates that the model was a good fit for the data at each education level.

**Figure 1:**
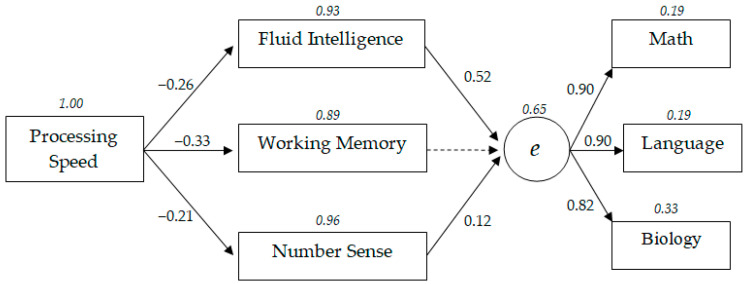
Model with relationships between cognitive abilities and general academic achievement in primary school education.

Figure 1 shows the model structure of the relationships between academic success and information processing speed, working memory, number sense, and fluid intelligence. The standardized structural coefficients (*p* < 0.05) obtained during the structural modeling in a sample of primary school education levels are shown. Dotted lines indicate nonsignificant relationships (*p* > 0.05). The residual dispersion highlighted by the italic font. The latent variable *e* is general academic achievement factor based on teachers’ grades in mathematics, Russian language, and biology.

As shown in Figure 1, the model considers general academic achievement as a latent variable based on three indicators of academic success: (1) mathematics, (2) Russian language, and (3) biology. At primary school ages, the success rates in academic disciplines (i.e., mathematics, Russian language, and biology) were almost equally loaded on the latent academic success factor “*e*” (from 0.82 to 0.90).

According to the model, the information processing speed is regarded as the basic cognitive characteristic underlying the higher-order cognitive indicators: fluid intelligence, working memory, and number sense. In turn, these cognitive indicators influence academic success. A comparison of the standardized structural coefficients reveals that at the primary level of education, the speed of information processing has a stronger influence on working memory (β = −0.33) and a weaker influence on fluid intelligence (β = −0.26) and number sense (β = −0.21). Regression weights between fluid intelligence, working memory, and number sense ranged from 0.27 to 0.35.

Results show that fluid intelligence is the strongest predictor of the general academic achievement at the primary level of schooling compared to other cognitive characteristics (β = 0.52).

Therefore, the most significant relationship between cognitive traits and academic success is the indirect influence of information processing speed through fluid intelligence, as confirmed by standardized path coefficients calculated according to the principles of structural equation modeling [13,36]. Thus, the regression weight of the path “Information processing speed–Fluid intelligence–General academic achievement” was −0.26 × 0.52 = −0.13. In comparison, the regression weight of the path “Information processing speed–Working memory–Fluid intelligence–General academic achievement” was significantly lower and equaled β = −0.06 (−0.33 × 0.34 × 0.52) due to the greater number of manifesto variables. The standardized structural coefficients of other possible paths were even lower. For example, for the path “Information processing speed–Number sense–General academic achievement”, β was equal to −0.03 (−0.21 × 0.12). Additionally, it should be noted that the model with a direct path from information processing speed to academic success has unsatisfactory fit indices.

According to Figure 1, the residual dispersion of general academic success is 0.65. Thus, 35% of the dispersion in general academic success is explained during primary education using analyzed cognitive characteristics.

**Figure 2:**
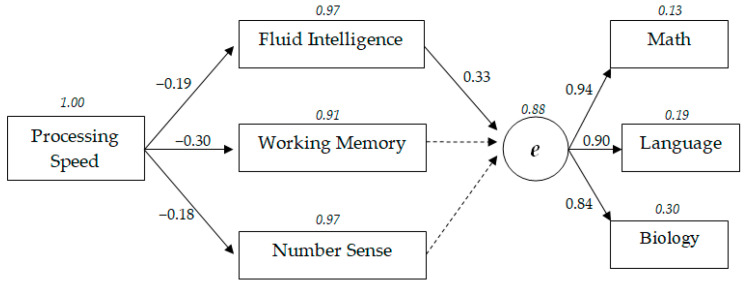
Model with relationships between cognitive abilities and general academic achievement in secondary school education.

Figure 2 presents a model of the structure of relationships between cognitive characteristics and academic success at the secondary level of school education. The standardized structural coefficients (*p* < 0.05) obtained during the course of structural modeling in a secondary-age sample are shown. Dotted lines indicate nonsignificant relationships (*p* > 0.05). The residual dispersion highlighted by the italic font. The latent variable *e* is general academic achievement factor based on teachers’ grades in mathematics, Russian language, and biology.

At the secondary level of school education, a comparative analysis of the standardized structural coefficients of the model shows that information processing speed has a greater influence on working memory (β = −0.30), a slightly weaker influence on the indices of fluid intelligence (β = −0.19) and number sense (β = −0.18). Regression weights between fluid intelligence, working memory, and number sense range from 0.28 to 0.30.

Indicators of success in academic disciplines (i.e., mathematics, Russian language, and biology) proved to be almost equally burdened by the latent factor of academic achievement “*e*” (from 0.84 to 0.90).

Fluid intelligence has the greatest influence among all cognitive characteristics on the general academic achievement factor at the secondary level of schooling (β = 0.33).

The most significant path from cognitive abilities to academic success is the trajectory of indirect influence of information processing speed through fluid intelligence on general academic success. In particular, the regression weight of the path “Information processing speed–Fluid intelligence–General academic achievement” was −0.19 × 0.33 = −0.06. In comparison to the described path, due to a greater number of manifesto variables, the regression weight of the path “Information processing speed–Working memory–Fluid intelligence–General academic achievement” was lower, β = −0.03 (−0.30 × 0.28 × 0.33). Other paths of cognitive abilities influencing the general academic success are not possible, as the contribution of working memory and number sense is not significant.

According to Figure 2, 12% of the dispersion of the general academic achievement at the secondary level of school education was explained by the analyzed cognitive abilities, as evidenced by the residual dispersion of 0.88.

**Figure 3:**
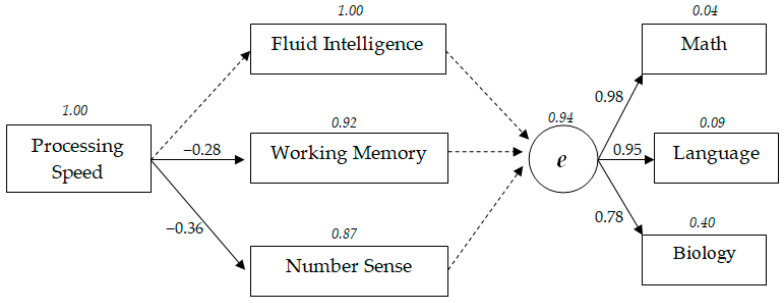
Model with relationships between cognitive abilities and general academic achievement in high school education.

Figure 3 presents a model of the structure of relationships between cognitive characteristics and academic success at a high level of school education. The standardized structural coefficients (*p* < 0.05) obtained in the course of structural modeling on the high school sample are indicated. Dotted lines indicate nonsignificant relationships (*p* > 0.05). The residual dispersion highlighted by the italic font. The latent variable *e* is general academic achievement factor based on teachers’ grades in mathematics, Russian language, and biology.

At the high level of school education, a comparative analysis of standardized structural coefficients of the model shows that the speed of information processing had a greater influence on number sense (β = −0.36), had a slightly weaker influence on the working memory (β = −0.28), and was not associated with fluid intelligence (*p* > 0.05). The standardized coefficient between fluid intelligence and working memory was 0.28, and that between number sense and working memory was −0.30. The relationship between fluid intelligence and number sense was not statistically significant (*p* > 0.05).

It should especially be noted that at the high level of education, none of the analyzed cognitive characteristics had a statistically significant impact on the academic success factor (*p* > 0.05).

Among the indicators of academic achievements, the most heavily loaded on the general academic success was the grade in mathematics (β = 0.98), followed by the grade in language (β = 0.95). The success rate in biology had the lowest loading (β = −0.78).

## 4. Discussion

This study analyzed the relationship between cognitive abilities and academic achievement across the whole schooling period. Indicators of cognitive ability (i.e., information processing speed, visuospatial working memory, number sense, and fluid intelligence) were considered predictors of general academic success, which was derived from school grades in mathematics, language, and biology.

Descriptive statistics showed different developmental trends in cognitive functioning and academic success across school education. In particular, cognitive characteristics improve from primary to high school education. In contrast, the grades awarded by teachers get lower as schoolchildren get older. Some improvements in teachers’ grades were observed at a high level of schooling as a result of the selection of schoolchildren. This divergent trend may be related to the reduced influence of cognitive ability on the development of individual differences in academic success throughout schooling [8,25,40].

The correlation analysis showed that fluid intelligence is most closely linked to academic success, which is fully consistent with previous research on the leading role of fluid intelligence in learning [1,3,5,20,26]. However, it should be noted that no significant relationship between academic success in language and biology and information processing speed was observed at any level of education, with the exception of a weak relationship between information processing speed and academic success in mathematics in primary school. The fact that there are no direct correlations, however, may indicate a more complex, indirect relationship between processing speed and academic achievement, which was further investigated using structural equation modeling. In addition, moderate correlations between cognitive characteristics were identified, allowing the analyzed cognitive characteristics to be considered predictors of academic success in further analysis. Conversely, close correlations between academic success indicators in mathematics, Russian language, and biology make it possible to analyze academic success as a generalized metric derived from grades in these school disciplines.

Structural modeling showed that throughout general education, the structure of relationships between cognitive characteristics and academic success remains stable. Information processing speed is a key predictor of higher-order cognitive characteristics (i.e., fluid intelligence, working memory, and number sense), which in turn contribute to overall academic success. A number of researchers also emphasized the importance of information processing speed as the basis for individual differences in higher-order cognitive characteristics, such as fluid intelligence [3,16]. These studies also noted the relationship between information processing speed and working memory [13,18,19]. The theoretical model of the structure of relationships between cognitive abilities and academic achievement, which best describes empirical data, matches to some extent the model used in the study of H. Rindermann and A. Neubauer [13]. Mirroring the findings of this study, they found the model that links information processing speed with higher-order cognitions (in particular, intelligence and creativity), which in turn influence academic success to be the best fit.

The results show that the relationships between cognitive characteristics and academic success differ at each level of schooling. In particular, the most significant relationship (at the primary and secondary levels of school education) is that between information processing speed and fluid intelligence, which in turn contributes to general academic achievement. However, at a high level of school education, no significant relationships between cognitive traits and academic success were found. In other words, in a sample of high school added students, the model being tested is split into two parts that are not related to each other: cognitive characteristics and academic success factors. This can be explained by the criteria for selecting students for further study in school: traditionally, more successful students continue to study at a high educational level. Consequently, the sample of high school education is selected and is leveled in terms of the development of cognitive characteristics. At all levels of school education, the most important predictor of general academic achievement is grades in mathematics (values of β up to 0.95), and the least important are grades in biology (values of β up to 0.82).

## 5. Conclusions

Thus, the study shows the structure of relationships between cognitive characteristics and academic achievement that is relevant during the whole schooling period. This structure is described by a single model that is relevant for all levels of school education: information processing speed is a key predictor of fluid intelligence, working memory, and number sense, which in turn contribute to individual differences in academic achievement.

Additionally, the specificity of the relationship between individual cognitive characteristics and academic success at each level of schooling was shown. Thus, in the period of compulsory school education (primary and secondary school education), the relationship between information processing speed and general academic success has the greatest regression weight, with fluid intelligence as the mediator. No statistically significant relationships between cognitive characteristics and academic success were found at the high level of school education. It was shown that the contribution of cognitive characteristics to individual differences in academic success decreases in the period from primary to complete level of general education, which may point toward the greater importance of motivational and personal predictors of academic success.

The study of the contribution of cognitive abilities to academic achievement will clarify the psychological mechanisms underlying individual differences in learning. A better understanding of the mechanisms of individual differences can form a foundation for developing personalized educational methods that can help improve the academic success of each student. Further directions of the project may be associated with conducting a longitudinal study and identifying early cognitive predictors of academic success at different levels of school education.

## Figures and Tables

**Table 1:** Descriptive statistics of cognitive abilities and academic success at primary, secondary, and high levels of school education.

Variable	Mean (Standard Deviation)
Fluid intelligence	37.99 (9.48) 45.62 (6.76) 51.42 (5.06)
Information processing speed	1.00 (0.32) 0.50 (0.20) 0.48 (0.20)
Visuospatial working memory	2.50 (1.86) 4.30 (1.85) 4.99 (1.80)
Number sense	95.48 (14.13) 104.20 (14.10) 109.60 (14.00)
Language	3.95 (0.60) 3.90 (0.60) 3.91 (0.68)
Math	4.06 (0.59) 3.80 (0.65) 3.94 (0.76)
Biology	4.48 (0.53) 3.97 (0.63) 4.04 (0.69)

**Table 2:** Correlations between cognitive abilities and academic achievement at primary (top line), secondary (middle line), and high (bottom line) levels of school education.

Variables	Language	Math	Biology
Processing speed	−0.02 −0.06 0.06	−0.09 * −0.06 0.04	−0.06 −0.04 0.01
Working memory	0.15 ** 0.11 ** 0.14 **	0.22 ** 0.11 * 0.10	0.17 ** 0.01 0.10
Number sense	0.24 ** 0.10 * 0.06	0.26 ** 0.13 ** 0.07	0,22 ** 0.04 0.10
Fluid intelligence	0.47 ** 0.33 ** 0.18 **	0.48 ** 0.36 ** 0.10	0.43 ** 0.27 ** 0.08

* *p* < 0.05, ** *p* < 0.01; top line—primary school, middle—secondary school, bottom—high school.

**Table 3:** Fit indices of Model 3 for primary, secondary, and high school education.

Indices	AIC	BIC	CFI	TLI	RMSEA	RMSEA_low_	RMSEA_high_
Primary	6647.17	−14125.86	0.996	0.991	0.027	0.000	0.055
Secondary	3304.24	−11505.64	0.997	0.993	0.030	0.000	0.066
High	995.50	−3274.59	1.006	1.013	0.000	0.000	0.000

AIC: Akaike information criterion, BIC: Bayesian information criterion, CFI: comparative fit index, TLI: Tucker–Lewis index, RMSEA: root mean square error of approximation, RMSEA_low_: the lower limit of the 95% confidence interval for RMSEA, RMSEA_high_: the upper limit of the 95% confidence interval for RMSEA.
